# A Pilot Feasibility Study of a Metronome-Cued mHealth System to Promote Walking Exercise in COPD

**DOI:** 10.3390/healthcare14131927

**Published:** 2026-07-01

**Authors:** Shuoshuo Wei, Yongfa Hao, Yi Zhang, Faxuan Wang, Ping Zhou, Wangshu Jiang, Yuanyuan Liu, Leiyi Sheng, Yuanyuan Jia, Yumin Zhou, Jiye An, Ning Deng, Juan Chen

**Affiliations:** 1 Ningxia Key Laboratory of Clinical and Pathogenic Microorganisms, Institute of Medical Sciences, Department of Respiratory and Critical Care Medicine, General Hospital of Ningxia Medical University, Yinchuan 750004, China; 18795399909@163.com (S.W.); haoyongfa@163.com (Y.H.); zhangyi517925@163.com (Y.Z.); zhouping0324@163.com (P.Z.); 18209601350@163.com (Y.J.); 2 Department of Pulmonary and Critical Care Medicine, The Affiliated Yijishan Hospital of Wannan Medical College, Wuhu 241001, China; 3 School of Public Health, Ningxia Medical University, Yinchuan 750004, China; faxuan203@163.com; 4 Department of Pulmonary Function, General Hospital of Ningxia Medical University, Yinchuan 750004, China; 5 Ministry of Education Key Laboratory of Biomedical Engineering, College of Biomedical Engineering and Instrument Science, Zhejiang University, Hangzhou 310027, China; orselchiang@163.com (W.J.); summersly0805@126.com (L.S.); an_jiye@zju.edu.cn (J.A.); 6 Department of Rehabilitation, General Hospital of Ningxia Medical University, Yinchuan 750004, China; 18695281759@163.com; 7 State Key Laboratory of Respiratory Disease, National Clinical Research Center for Respiratory Disease, Guangzhou Institute of Respiratory Health, The First Affiliated Hospital of Guangzhou Medical University, Guangzhou 510120, China; zhouyumin410@126.com

**Keywords:** chronic obstructive pulmonary disease, home-based rehabilitation, metronome-cued walking mHealth system, walking exercise

## Abstract

**Highlights:**

**What are the main findings?**

- After 12 weeks, the intervention group showed a mean increase in 6MWD of 35.32 m (95% CI: 27.84–42.80, *p* < 0.001), with 68% of participants achieving the minimum clinically important difference of +35 m.
- Significant improvements were also observed in the intervention group across all secondary outcomes, including the CAT, the CCQ, the mMRC dyspnea scale, and the HADS, indicating broad benefits in terms of symptom burden, quality of life, and emotional health.

**What are the implications of the main findings?**

- This metronome-cued walking program supported by mHealth technology offers a practical, scalable, and safe strategy for home-based pulmonary rehabilitation, addressing common barriers such as access to supervised center-based programs.
- The personalized, exercise-capacity-adjusted approach supports the integration of digital health tools into routine COPD management, potentially enabling more individualized care and improving long-term adherence to physical activity.

**Abstract:**

**Objectives:** This study was conducted to develop and evaluate a personalized, home-based metronome-cued walking mHealth system, tailored to individual exercise capacity, and assess its effects on physical activity and quality of life in patients with chronic obstructive pulmonary disease (COPD). **Methods:** A single-center prospective study was conducted in 40 patients with stable COPD, who were assigned to an intervention group (IG, *n* = 28) and a control group (CG, *n* = 12). The IG received a 12-week metronome-cued walking training through the mobile health system. The initial walking intensity was set at 70% of the patient’s baseline level, as determined by the 6 min walking distance (6MWD) and cardiopulmonary exercise testing (CPET). The duration of the training was adjusted according to the Borg scale score and was conducted at least three times per week. The CG received standard care, including lifestyle advice, medications and dietary guidance. Outcomes, including the 6MWD, the COPD Assessment Test (CAT), the Clinical COPD Questionnaire (CCQ), the modified Medical Research Council (mMRC) dyspnea scale, and the Hospital Anxiety and Depression Scale (HADS), were recorded at baseline and week 12. **Results:** Among 40 enrolled patients, the IG showed improvements compared with the CG. The mean 6MWD increased by 35.32 m (95% CI: 27.84–42.80, *p* < 0.001) in the IG, with 68% of participants achieving the minimum clinically important difference (MCID) of +35 m. The CAT, the CCQ, the mMRC dyspnea scale, and the HADS also improved in the IG. These findings suggest that the intervention may improve exercise capacity, dyspnea, health-related quality of life and psychological well-being in this patient population. No intervention-related adverse events were observed during the study period. **Conclusions:** In this small, non-randomized pilot study, a personalized, home-based metronome-cued walking program supported by mHealth technology was feasible and showed preliminary signals of benefit for exercise capacity, dyspnea, quality of life and psychological well-being in patients with COPD.

## 1. Introduction

Chronic obstructive pulmonary disease (COPD) remains one of the most prevalent chronic respiratory diseases worldwide, affecting over 400 million people and representing the fourth leading cause of death globally [1]. The disease is characterized by persistent respiratory symptoms and progressive airflow limitation, which significantly impair patients’ exercise capacity and health-related quality of life [2]. Beyond its respiratory manifestations, COPD frequently coexists with cardiovascular, metabolic, and psychological conditions, creating a complex clinical picture that magnifies disability and complicates treatment decisions [1]. Patients with COPD commonly report dyspnea as their most distressing symptom during physical activity, initiating a vicious cycle of physical inactivity, muscle deconditioning, and further exercise intolerance.

Pulmonary rehabilitation (PR) is a cornerstone of the non-pharmacological management of COPD. Comprehensive PR programs, including exercise training, education, and behavioral interventions, have been shown to alleviate dyspnea, improve exercise tolerance, enhance quality of life, and reduce risks of exacerbation [3,4]. The American Thoracic Society/European Respiratory Society guidelines strongly recommend PR as a standard intervention for patients with moderate-to-severe disease [5]. However, despite these well-documented benefits, PR remains dramatically underused. Recent studies show that fewer than 5% of Medicare beneficiaries hospitalized with COPD enroll in PR, and more than half of US counties lack a hospital-based outpatient program [6]. Common barriers include transportation difficulties, financial constraints, limited accessibility in rural areas, and inadequate healthcare provider competencies [7]. These limitations have created an urgent need for effective home-based alternatives that can overcome access barriers while maintaining therapeutic efficacy [8].

Home-based pulmonary rehabilitation (HBPR) has emerged as a promising complement to traditional center-based programs, particularly during the COVID-19 pandemic, when access to facility-based rehabilitation was severely restricted [9]. A recent scoping review of 44 studies reported that telemonitoring and telerehabilitation interventions led to improvements in health-related quality of life in 84% of studies compared to pre-intervention levels and were as effective as standard care [10]. Moreover, participants in HBPR programs were more likely to complete rehabilitation than those in center-based programs [8,11]. Recent evidence suggests that mobile health (mHealth)-enhanced PR can achieve equivalent or even superior improvements in health status compared to center-based PR, with superior improvements in COPD Assessment Test (CAT) scores and comparable gains in exercise capacity [12].

Walking is a fundamental and accessible form of physical activity for patients with COPD. Rhythmic auditory stimulation, such as metronome cueing, represents an innovative approach for optimizing gait patterns and regulating walking speed during exercise training [13]. The underlying mechanism involves strong sensorimotor coupling between auditory rhythm and the motor system, which can entrain movement without requiring conscious cognitive effort [13]. Previous research has demonstrated that metronome-paced walking can improve 6 min walk distance, improve breathing patterns, and increase oxygen saturation during exercise in patients with COPD [14]. Pomidori et al. found that home exercise training performed at a pace determined by a metronome resulted in greater improvements in 6 min walk distance compared to walking a known distance in a fixed time, suggesting that metronome cueing helps patients achieve and sustain optimal exercise intensity [15]. Furthermore, Bernardi et al. [14] demonstrated that a 12-week home-based, moderate-intensity exercise program using a metronome significantly improved ventilatory efficiency and reduced dyspnea perception during exercise.

Despite these promising findings, the integration of metronome-cued walking into personalized, home-based mHealth systems has rarely been investigated. The combination of individualized exercise prescription based on baseline functional capacity, real-time auditory guidance via mHealth technology, and structured home-based training may offer a comprehensive approach to COPD management that addresses both physical and psychological dimensions of the disease [16,17].

Therefore, in this study, we aimed to develop and evaluate a personalized, home-based metronome-cued walking mHealth system tailored to individual exercise capacity, as well as to assess its effects on physical activity and quality of life in patients with COPD.

## 2. Materials and Methods

### 2.1. Study Design

This study was a single-center, prospective, non-randomized controlled study with a 12-week intervention period. The study was conducted from October 2020 to September 2021 at the Department of Respiratory and Critical Care Medicine, General Hospital of Ningxia Medical University, a tertiary medical center with 3500 beds in Ningxia, China. The study protocol was approved by the Ethics Committee of the General Hospital of Ningxia Medical University (No. 2020-338) and conducted in accordance with the Declaration of Helsinki. Written informed consent was obtained from all participants prior to enrollment.

### 2.2. Participants

Patients with stable COPD were recruited from the outpatient clinic. Eligibility was determined by respiratory medicine specialists. The inclusion criteria were as follows: (1) a confirmed diagnosis of COPD based on pulmonary function testing (post-bronchodilator forced expiratory volume in 1 s (FEV_1_)/forced vital capacity (FVC) < 0.70; FEV_1_ < 80% predicted); (2) age ≥ 18 years; (3) the ability to independently operate a smartphone after training, with adequate hearing and vision; and (4) provision of a signed informed consent form. The exclusion criteria included musculoskeletal abnormalities, dizziness, substantial sensory or motor impairments, dementia, severe cardiovascular disease, or other comorbidities that could impede participation in the walking exercise program.

Eligible patients who agreed to participate were assigned to either the intervention group (IG, *n* = 38) or the control group (CG, *n* = 16) based on their preference. All participants received standard medical care throughout the study period. As a pilot feasibility study, neither a formal power calculation nor a formal sample size calculation based on a predetermined effect size was performed. Instead, the sample size (*n* = 54) was pragmatically determined considering both the recruitment capacity of the single center over the study period (12 weeks) and the feasibility of delivering the personalized mHealth intervention.

### 2.3. Intervention Protocol

The walking intervention was prescribed for a duration of 12 weeks, with a minimum of 3 sessions per week. Each session initially lasted 30 min and was progressively extended to a maximum of 60 min, tailored to the individual patient’s exercise tolerance and clinical feedback.

#### 2.3.1. Intervention Group: Metronome-Cued Walking mHealth System

**Figure 1:**
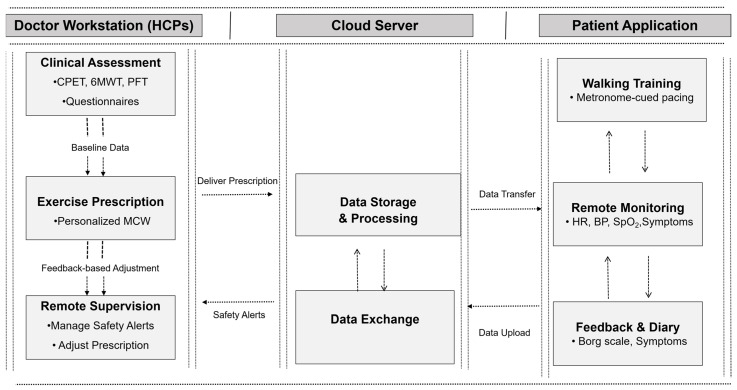
The overall architecture of the proposed system.

Participants in the IG were enrolled in a home-based walking program supported by a personalized metronome-cued walking mHealth system (developed in collaboration with Zhejiang University, Hangzhou, China), developed in collaboration with Zhejiang University [18]. As illustrated in Figure 1, the proposed system adopts a three-tier architecture comprising a Doctor Workstation (for healthcare professionals (HCPs)), a Cloud Server, and a Patient Application. First, HCPs conduct clinical assessments (including CPET (MasterScreen CPET, Erich Jaeger GmbH, Hoechberg, Germany), 6MWT, PFT (MasterScreen COPD, Erich Jaeger GmbH, Hoechberg, Germany), and questionnaires) to collect patients’ baseline data. Based on these data, a personalized exercise prescription (e.g., metronome-cued walking (MCW)) is generated and delivered to the Patient Application via the Cloud Server. During the walking training, the Patient Application actively performs remote monitoring by collecting vital signs (e.g., HR, BP, SpO_2_ (Yuwell, Jiangsu Yuyue Medical Equipment & Supply Co., Ltd., Danyang, China)) and symptoms, along with subjective feedback (e.g., Borg scale) via e-diaries. These data are seamlessly uploaded to the Cloud Server for storage, processing, and data exchange. Crucially, if abnormal physiological data are detected, the Cloud Server automatically transmits safety alerts to the Doctor Workstation. This closed-loop mechanism allows HCPs to maintain remote supervision of patients and make timely, feedback-based adjustments to the exercise prescription, thereby ensuring both the safety and efficacy of the rehabilitation process (Figure 1 and Table 1).

The platform consists of three main components: the Doctor Workstation for clinical assessment, exercise prescription, and remote supervision; the Cloud Server for data storage, processing, and exchange; and the Patient Application for walking training guidance, remote monitoring, and patient feedback. The arrows indicate the direction of data transmission and the closed-loop feedback mechanism. Horizontal dotted arrows across columns represent real-time data transmission and command delivery between the doctor workstation, cloud server, and patient application. Vertical dashed arrows within the “Doctor Workstation” column denote sequential clinical workflows (from clinical assessment to prescription formulation, and feedback-based adjustment). Bidirectional vertical dashed arrows columns indicate continuous data exchange, real-time feedback loops, and daily cycling among walking training, remote monitoring, and diary feedback.

Assessment Module: Baseline assessments included cardiopulmonary exercise testing (CPET), pulmonary function testing [19], the 6 min walking test (6MWT) [20], the COPD Assessment Test (CAT) [21], the modified Medical Research Council (mMRC) dyspnea scale [22], the Hospital Anxiety and Depression Scale (HADS) [23], and the Clinical COPD Questionnaire (CCQ) [24].

Prescription Module: Rehabilitation specialists created individualized walking exercise prescriptions based on baseline assessments. The prescription covered exercise intensity, frequency and duration. A built-in metronome provided rhythmic auditory stimulation to regulate walking speed. Walking speed (m/min) was calculated using the formula, namely Walking speed = (VO_2_ − 3.5)/0.1 [25], based on oxygen consumption derived from CPET. Stride frequency (steps/min) was calculated as: Stride frequency = Walking speed / Step distance. CPET was used to estimate individual exercise capacity and to determine the prescribed walking speed. The initial target walking speed was derived from CPET-estimated VO_2_ and set at 70% of the maximal baseline speed. Subsequent adjustments to exercise duration were based on Borg dyspnea scores.

Feedback Module: Patients reported symptoms such as dyspnea and fatigue after each exercise session, enabling remote monitoring by healthcare professionals.

Safety Supervision Module: Key parameters, including heart rate, blood pressure, oxygen saturation, and symptoms, were monitored. Automated alerts were triggered if abnormal values were detected.

**Figure A1:**
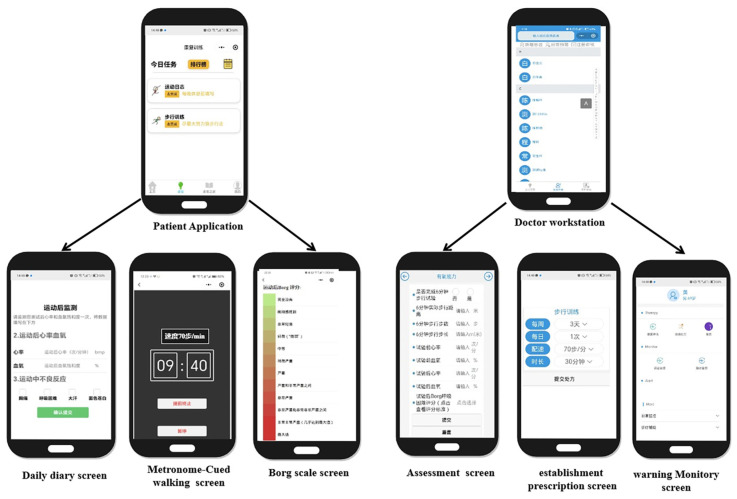
The remote deployment and home management of personalized walking prescriptions in mobile applications.

The mobile application consisted of three components: a doctor interface, cloud storage, and a patient interface. After enrollment, doctors uploaded personalized walking prescriptions to the application (Appendix A Figure A1). Patients performed the prescribed walking exercises at home at the set time and duration.

Exercise intensity was monitored using the Borg scale (0–10) for perceived exertion during each session [26]. Walking duration was adjusted based on Borg scores:

Score ≥ 7: walking time reduced by 6 min.

Score 4–6: walking time unchanged.

Score < 4: walking time increased by 6 min.

As illustrated, the overall intervention spanned 12 weeks. During daily practice, a closed-loop interaction was established: the system automatically dispensed the exercise prescription to the Patient Application, while the patient’s daily exercise data and safety alerts were uploaded to the Cloud Server for the supervisor to review using the Doctor Workstation. In terms of daily monitoring and dynamic adjustment, patients were required to perform a pre-exercise check (including SpO_2_ and heart rate). Subsequently, they initiated the metronome-cued walking at an initial pace of 70% of their baseline maximum speed. Crucially, the walking duration was dynamically adjusted based on the patient’s perceived exertion. After each session, patients rated their dyspnea using the Borg scale (0–10). If the score was <4, the exercise duration for the next session was increased by 6 min (up to a maximum of 60 min). If the score was between 4 and 6, the current duration was maintained. If the score was ≥7, the duration was decreased by 6 min. Finally, all post-exercise data were uploaded to allow the supervisor to perform a safety review.

**Figure 2:**
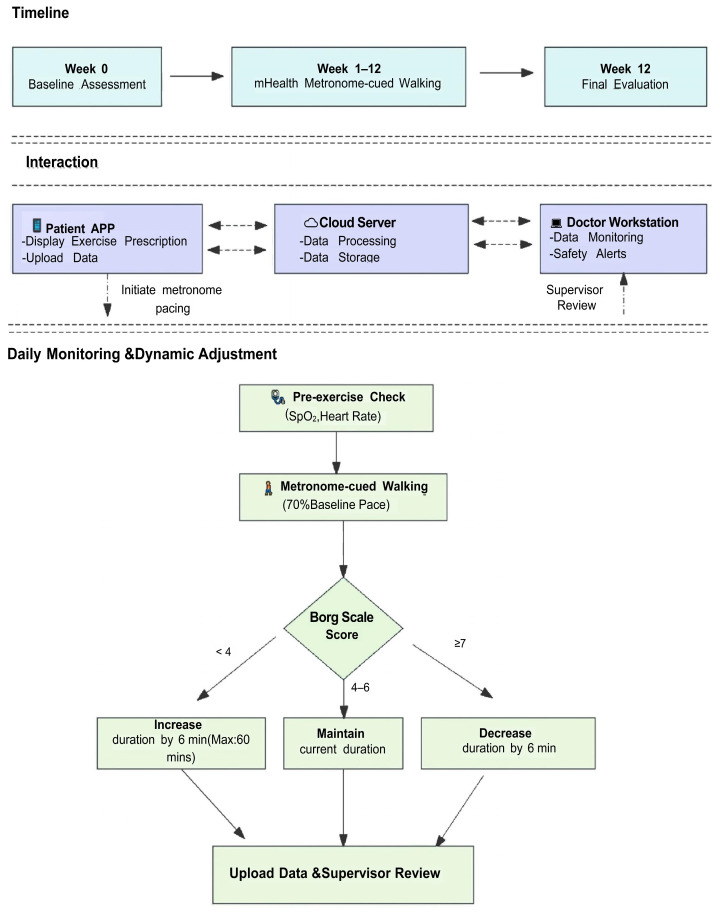
Flowchart of the mHealth-based metronome-cued walking intervention. The framework illustrates the intervention process across three dimensions. The timeline displays the 12-week study duration from baseline to the final evaluation. The interaction panel depicts the data flow among the Patient Application, Cloud Server, and Doctor Workstation, highlighting the delivery of the exercise prescription and the review performed by the clinical supervisor. The daily monitoring and dynamic adjustment section details the patient’s daily routine: conducting pre-exercise vital sign checks (SpO_2_ and heart rate), engaging in metronome-cued walking at 70% of the baseline pace, and dynamically adjusting the exercise duration (±6 min) based on the Borg Dyspnea Scale score, followed by uploading data for the supervisor to review.

A detailed workflow of the mHealth intervention, including the timeline, system interaction, and daily dynamic adjustment, is illustrated in Figure 2.

#### 2.3.2. Control Group (CG)

Participants in the CG received standard care, including routine medical follow-up, lifestyle advice, medication management, and dietary guidance, without being subject to the mHealth-based walking intervention.

#### 2.3.3. Adherence and Remote Monitoring

Adherence to the metronome-cued walking program was monitored through the mHealth system, which automatically recorded each training session initiated by participants in the IG. For each session, the system logged the date, walking duration, and Borg scale scores reported upon completion. A training session was considered “compliant” when participants completed the prescribed walking duration while maintaining the recommended step rate synchronized with the metronome. Real-time remote monitoring was enabled via the system’s Doctor Workstation, allowing the research team to review each participant’s training logs on a weekly basis. If no training record was detected for three consecutive days, a research assistant contacted the participant by phone to ensure engagement. The adherence rate was defined as the number of completed training sessions divided by the total number of prescribed sessions (36 sessions, based on 3 sessions per week over 12 weeks).

### 2.4. Outcome Measures

All outcome assessments were conducted at baseline and at week 12 by trained assessors blinded to group allocation.

Primary outcome:

Exercise capacity was assessed using the 6 min walking distance (6MWD). The test was performed in a 30 m indoor corridor by trained personnel, strictly following the clinical practice guidelines of the American Thoracic Society (ATS) [20]. A positive response was defined as an improvement in 6MWD exceeding the minimum clinically important difference (MCID) of +35 m [27].

Secondary outcomes:

Health-related quality of life was assessed using the COPD Assessment Test (CAT) and the Clinical COPD Questionnaire (CCQ). Specifically, we used the official simplified Chinese version of the CAT (available at www.catestonline.org); the CCQ followed the official Chinese version (www.ccq.nl).

Dyspnea severity was assessed using the modified Medical Research Council (mMRC) dyspnea scale. The mMRC dyspnea scale utilized the standard descriptions from the Guidelines for the Diagnosis and Treatment of COPD in China (2021 Revision) [28].

Psychological status was assessed using the Hospital Anxiety and Depression Scale (HADS) (specifically the Chinese version validated by Leung et al. [29]).

All questionnaires were administered in Chinese and self-completed by participants whenever possible. For those with literacy or comprehension difficulties, trained researchers provided neutral interview assistance and verified the completeness of the responses without influencing the participants’ choices.

Safety was ensured by recording the incidence of intervention-related adverse events throughout this study.

### 2.5. Statistical Analysis

Statistical analyses were performed using SPSS version 23.0 (IBM, Armonk, NY, USA). Graphs were created using GraphPad Prism version 8.4.0 (GraphPad Software, San Diego, CA, USA). Continuous variables with a normal distribution were expressed as mean ± standard deviation (SD); non-normally distributed variables were reported as median (interquartile range). Categorical variables were compared using the Pearson χ^2^ test or Fisher’s exact test, as appropriate. Within-group comparisons (baseline vs. week 12) were analyzed using the paired *t*-test for normally distributed data or the Wilcoxon signed-rank test for non-normally distributed data. Between-group differences in changes from baseline were analyzed using independent t-tests or the Mann–Whitney U tests, as appropriate. A two-tailed *p*-value < 0.05 was considered statistically significant.

To minimize potential confounding and selection bias inherent to the non-randomized, self-selected design, an analysis of covariance (ANCOVA) was performed on the primary outcome (6MWD at 12 weeks). Group (intervention vs. control) was entered as the fixed factor, while baseline 6MWD, age, and FEV1% predicted were adjusted as covariates. The estimated marginal means, adjusted mean differences, 95% confidence intervals (CIs), and partial eta-squared were calculated.

All analyses were conducted on the completer population, defined as participants who completed the 12-week follow-up assessment. Accordingly, this study did not employ an intention-to-treat approach, and missing post-intervention data were not imputed.

## 3. Results

### 3.1. Participant Disposition

**Figure 3:**
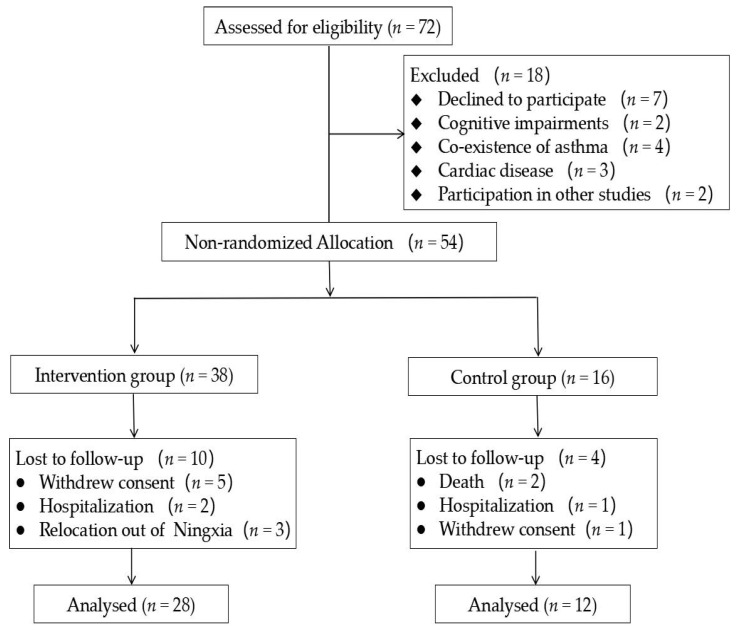
A participant flow diagram.

A total of 72 patients with stable COPD were assessed for eligibility. Of these, 18 were excluded for the following reasons: seven declined to participate, two had cognitive impairments, four had coexisting asthma, three had cardiac disease, and two were already enrolled in other studies. A total of 54 participants were initially enrolled. During the 12-week study period, 14 participants were lost to follow-up or had missing data due to hospitalization (*n* = 3), withdrawal of consent (*n* = 6), relocation out of Ningxia (*n* = 3), or death (*n* = 2). Finally, 40 participants completed the study and were included in the final analysis: 28 in the intervention group (IG) and 12 in the control group (CG). A participant flow diagram is presented in Figure 3. The 14 participants who did not complete the study were not included in the primary outcome analyses because post-intervention data were missing. Given the pilot nature of the study, no imputation methods were applied for missing data, and only completers were analyzed.

### 3.2. Baseline Characteristics

The baseline demographic and clinical characteristics of the study participants are summarized in Table 2. All participants were male. There were no significant differences between the two groups at baseline in terms of age, body mass index (BMI), pulmonary function (FEV1% predicted, FVC% predicted), exercise capacity (6MWD), symptom scores (CAT, CCQ, mMRC), psychological status (HADS) or BODE index (all *p* > 0.05). Regarding medication use, the most common treatments were long-acting β2 agonists (LABAs), long-acting muscarinic antagonists (LAMAs), and inhaled corticosteroids (ICSs). The proportion of participants using these medications, as well as long-term oxygen therapy, was similar between the two groups. These findings indicate that the two groups were well-matched at baseline.

### 3.3. Effectiveness of Home-Based Walking Training

After the 12-week intervention, significant improvements were observed in the IG compared to the CG across multiple outcome measures.

#### 3.3.1. Exercise Capacity

Exercise capacity, assessed by the patients’ 6MWD, increased significantly in the IG, with a mean improvement of 35.32 m (95% CI: 27.84 to 42.80). In contrast, a slight decrease was observed in the CG (mean change: −8.50 m; 95% CI: −17.52 to 0.52). The between-group difference in 6MWD change was statistically significant (*p* < 0.001). After adjusting for age, FEV1% predicted, and baseline 6MWD using ANCOVA, the intervention group still achieved a significantly higher 6MWD at 12 weeks compared to the control group, with an estimated marginal mean of 497.26 m (95% CI: 490.15 to 504.36) versus 453.24 m (95% CI: 442.36 to 464.12). The adjusted mean difference between the two groups was 44.02 m (95% CI: 30.99 to 57.05, F = 47.038, *p* < 0.001, Partial Eta^2^ = 0.573), demonstrating a robust and clinically meaningful treatment effect (Table 3).

#### 3.3.2. Quality of Life and Symptom Scores

Health-related quality of life and symptom scores also improved significantly in the IG relative to the CG:

CAT score: −2.21 vs. +0.33 (*p* = 0.004);

CCQ score: −0.13 vs. +0.10 (*p* = 0.007);

mMRC dyspnea scale: −0.54 vs. 0.00 (*p* = 0.014).

#### 3.3.3. Psychological Status

Psychological symptoms improved significantly in the IG compared to the CG:

HADS-Anxiety score: −1.11 vs. +0.92 (*p* = 0.001);

HADS-Depression score: −1.18 vs. +0.17 (*p* = 0.031).

#### 3.3.4. BODE Index

No significant difference was observed in the BODE index between the two groups after the intervention (*p* > 0.05).

Detailed results for all outcome measures are presented in Table 4.

### 3.4. Response to 6MWD in the Intervention Group

**Figure 4:**
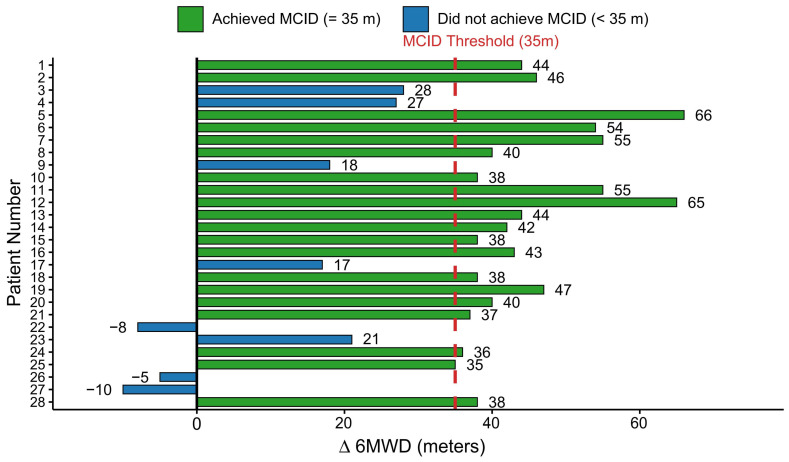
Individual changes in 6 min walk distance (Δ6MWD).

Among the 28 participants in the IG, 19 (67.86%) achieved a clinically significant improvement in 6MWD that exceeded the minimum clinically important difference (MCID) of 35 m, with a maximum improvement of 65 m. One participant (3.57%) improved by exactly 35 m, and five participants (17.86%) showed improvement below the MCID. Three participants (10.71%) experienced a decrease in 6MWD after the 12-week intervention. This distribution of 6MWD changes is illustrated in Figure 4.

The red dashed line represents the minimal clinically important difference (MCID) threshold of 35 m. Green bars indicate patients who achieved or exceeded the MCID, while blue bars indicate those who did not. Exact change values are shown at the end of each bar.

### 3.5. Adherence

The overall mean adherence rate in the IG was 84.2% (range: 58.3–100%). Regarding application usage, participants logged a median of 32.5 active days (interquartile range: 28–35) over the 12-week period, with an average of 2.8 completed sessions per week. The average duration of each exercise session was 36.4 ± 11.2 min. No participant was lost to follow-up due to technology-related difficulties. These data indicate that the mHealth system achieved satisfactory real-world engagement and that remote monitoring was feasible in this COPD population.

### 3.6. Safety

The Metronome-Cued Walking mHealth System was well tolerated, and no serious adverse events (SAEs) were reported during the study period. A total of five (*n* = 5) mild, non-serious adverse events occurred during the initial week of training, including transient lower-limb muscle soreness (*n* = 2) and mild fatigue (*n* = 3). All symptoms resolved spontaneously within 24 h and were assessed by the study physician as normal physiological adaptations rather than a safety issue. The monitoring module of the mHealth system was programmed to detect physiological abnormalities based on five predefined warning thresholds. Across all logged home exercise sessions, no safety alerts were triggered. Consequently, no exercise sessions were suspended, and no walking prescriptions were modified or discontinued for safety reasons during the 12-week study period.

## 4. Discussion

In this study, we developed and evaluated a personalized, home-based metronome-cued walking mHealth system for patients with stable COPD. The results suggested that this 12-week intervention was associated with improvements in exercise capacity, alleviated dyspnea, and enhanced both health-related quality of life and psychological well-being. Specifically, the intervention group achieved a mean increase of 35.32 m in 6 min walk distance (6MWD), with 68% of participants exceeding the minimum clinically important difference (MCID) of +35 m. These preliminary results suggest that the mHealth system may be a feasible adjunctive tool for COPD home-based management, which warrants further validation in larger, randomized studies.

### 4.1. Improvement in Exercise Capacity

The observed 35.32 m improvement in 6MWD in our study exceeds the recently established MCID for exercise interventions in respiratory conditions, which a comprehensive meta-analysis of 42 studies involving 13,949 participants estimated to be between 24 and 26 m [30]. These preliminary findings are within the range reported for various exercise modalities in COPD. A network meta-analysis performed by Xie et al. demonstrated that continuous aerobic training, interval training, and resistance training all significantly improved 6MWD, with mean differences ranging from 33.3 to 84.5 m [31]. The 35.32 m improvement in our study is comparable to the effects of qigong (33.3 m) and continuous aerobic training (55.2 m), suggesting that metronome-cued walking may have effects comparable to these established interventions.

The observed improvements could be partly attributed to the rhythmic auditory cueing provided by the metronome. Research has shown that metronome-paced walking can optimize gait patterns and regulate walking speed, potentially reducing the oxygen cost of walking and improving exercise efficiency in patients with COPD [31]. This mechanism aligns with findings that interval training, which involves structured alternation between high and low intensity, ranked highest for improving 6MWD among exercise modalities [14]. Our system, which allowed real-time adjustments of training duration based on Borg scale scores, might similarly offer interval-like benefits while maintaining individualized exercise prescription.

Despite the overall positive outcomes, we observed inter-individual variability in response: approximately 28.6% of participants in the intervention group (8 out of 28) did not achieve the MCID for 6MWD, with three of them (10.7%) experiencing a decline in walking distance. Such variation is well-documented in exercise-based COPD interventions. We identified several potential contributing factors, including baseline functional status (where a ‘ceiling effect’ may limit gains in patients with higher initial 6MWD) [3], psychological barriers (specifically dyspnea-related kinesiophobia, which can restrict exercise intensity) [32], and adherence variability among participants. While our current sample size precludes a robust subgroup analysis to statistically identify these predictors, we have highlighted this as an area for future research. Larger-scale studies should systematically explore baseline characteristics, such as frailty, nutritional status, and psychological resilience, to better distinguish responders from non-responders in metronome-cued walking interventions.

### 4.2. Impact on Physical Activity and Quality of Life

The improvements in the CAT, the CCQ, and the mMRC scores in our intervention group reflect potential enhancements in both symptom burden and health-related quality of life. These findings are consistent with a large body of evidence demonstrating that home-based pulmonary rehabilitation programs can achieve outcomes comparable to center-based programs [8,11]. A recent scoping review of 44 studies reported that telemonitoring and telerehabilitation interventions led to improvements in health-related quality of life in 84% of studies compared to pre-intervention levels, as well as that they were as effective as standard care [10].

The integration of physical activity monitoring with goal setting and health coaching, as implemented in our system, aligns with the “treatable traits” paradigm for chronic airways disease management. A recent systematic review and meta-analysis of 236 studies (25,278 participants) identified physical inactivity as a highly prevalent and clinically relevant treatable trait in COPD, with daily step counts significantly associated with 6MWD and mMRC scores [33]. The authors concluded that physical activity satisfies the core criteria for a treatable trait—being clinically relevant, measurable, and treatable—supporting its inclusion in routine disease evaluation and management [33].

### 4.3. Psychological Benefits

The improvement in Hospital Anxiety and Depression Scale (HADS) scores in our intervention group highlights a potential psychological benefit of the metronome-cued walking program. These findings are supported by recent meta-analyses demonstrating that mind–body exercise interventions significantly reduce anxiety and depression in elderly COPD patients, with standardized mean differences of −0.59 and −0.34, respectively [34]. While our intervention was not designed as a mind–body exercise, the structured nature of metronome-cued walking combined with mHealth feedback might provide similar psychological benefits through enhanced self-efficacy, reduced dyspnea-related fear, and increased sense of control over the exercise process.

### 4.4. Adherence and Safety

The absence of intervention-related serious adverse events in our study provides preliminary evidence of the short-term safety of the metronome-cued walking program when appropriately prescribed based on individual exercise capacity. Adherence to home-based exercise programs in COPD can be challenging, but our findings suggest that the combination of personalized prescription, real-time feedback, and mHealth support might facilitate sustained engagement. Research by Cerini et al. on a home-based exercise program (HOMEX) found that 70% of participants continued with training until study end, with self-perceived improvement in strength and integration of training into daily routine identified as key facilitators of long-term motivation [35]. These insights are broadly consistent with our system design, which emphasizes individualized exercise prescription and integration of training into daily activities.

### 4.5. Comparison with Digital Health Interventions

Our metronome-cued walking mHealth system can be classified as a composite digital intervention combining a mobile application with physiological feedback (App + Sensor). A recent network meta-analysis comparing various digital health intervention modalities found that composite digital interventions ranked second (SUCRA = 66.8%) for improving daily step counts, while wearable only ranked highest for improving 6MWD (SUCRA = 90.3%) [36]. However, the authors noted that the certainty of evidence was low, and digital health interventions should be viewed as complementary rather than full replacements for traditional rehabilitation [36]. Our pilot results are consistent with this perspective, suggesting that personalized, home-based interventions may help extend the reach of pulmonary rehabilitation.

From an economic perspective, telerehabilitation has been shown to be a cost-effective alternative to center-based pulmonary rehabilitation, with an internal rate of return of 134% over five years [37]. Burge et al. reported that program completion was associated with a significant reduction in total healthcare costs in the following 12 months, regardless of whether rehabilitation was delivered via telerehabilitation or center-based models [37]. These findings suggest possible economic benefits of scaling up home-based programs, though this requires further investigation in our specific context.

### 4.6. Clinical Implications

If confirmed in larger trials, the findings of this pilot study may have several clinical implications. First, the metronome-cued walking approach provides a simple, reproducible method for regulating exercise intensity during home-based walking, which could address a key challenge in unsupervised rehabilitation. Second, the integration of mHealth technology enables real-time monitoring and feedback, potentially facilitating early detection of adverse responses and promoting safe exercise progression. Third, the personalized prescription based on baseline 6MWD and CPET data ensures that exercise intensity is appropriately tailored to individual functional capacity, which may enhance both safety and efficacy.

Given the high prevalence of physical inactivity in COPD and its association with adverse outcomes [33], interventions that might promote physical activity while maintaining safety are urgently needed. Our system offers a potential strategy that could be tested in various healthcare settings, which might eventually expand access to pulmonary rehabilitation for patients who face barriers to center-based programs.

### 4.7. Limitations

Several limitations should be acknowledged. First, in this study, we utilized a non-randomized, single-center prospective design. The allocation was partially influenced by patient preference, which may introduce selection bias. Although we performed ANCOVA to adjust for key baseline covariates (including age, lung function, and baseline exercise capacity) and confirmed that the treatment effect remained robust, potential unmeasured confounders may still exist. Future randomized controlled trials with larger cohorts are warranted to validate our findings. Participants highly motivated to use mHealth tools might differ from those opting for standard care regarding health literacy or self-management beliefs, potentially confounding the subjective outcomes (CAT, CCQ, mMRC, and HADS). Second, the relatively small sample size (*n* = 40) and the unbalanced group distribution (28 vs. 12) may limit the statistical power and increase the risk of Type II errors. Consequently, this study should be viewed as a pilot investigation, and the findings require validation through larger-scale, multicenter randomized controlled trials (RCTs). Third, our study population consisted entirely of male participants. This gender distribution was not a result of intentional selection bias, as patients were enrolled based on their willingness and strict inclusion/exclusion criteria without gender-based restrictions. Instead, this reflects the epidemiological characteristics of COPD in China and specifically in the Ningxia region. According to national surveys, the prevalence of COPD is significantly higher in men (11.9%) than in women (5.4%) [38], and in Ningxia, the prevalence among males (13.0%) is also markedly higher than in females (5.4%) [39]. While our cohort reflects the real-world recruited population at our center, the lack of female participants may limit the generalizability of the findings to all genders. Future multi-center studies should aim to include more female patients to verify the universal efficacy of the mHealth system. Fourth, we acknowledge that employing a “standard care only” control group, rather than an active control, is a limitation that may have amplified the observed between-group differences. Participants in the control group were managed with standard therapy and lifestyle counseling but lacked a supervised exercise regimen. Although this comparison provides some indication of the program’s potential clinical utility, the multifaceted nature of the intervention—comprising individualized exercise prescription, metronome pacing, and remote monitoring—precludes us from isolating the independent effect size of the mHealth platform itself. We recognize that factors such as enhanced patient engagement, regular follow-up contacts, and expectancy effects (the Hawthorne effect) within the intervention group may have contributed to the treatment outcomes. Therefore, we emphasize the need for future randomized controlled trials utilizing active control groups to more precisely delineate the specific efficacy of metronome-cued walking. Fifth, the study duration of 12 weeks does not allow assessment of the long-term sustainability of the observed improvements. Sixth, while the metronome-cued walking approach is innovative, the mechanisms underlying its benefits, particularly whether rhythmic auditory cueing directly improves gait efficiency or primarily enhances adherence, remain to be elucidated. Seventh, our analyses were conducted on the completer population only, and no intention-to-treat analysis was performed because post-intervention outcome data were unavailable for the 14 participants who discontinued the study (25.9% of the enrolled sample). The absence of imputation for missing data may have introduced attrition bias and affected the precision of the estimated intervention effects. Future larger trials should incorporate an intention-to-treat analysis with appropriate strategies for handling missing data. Finally, the cost-effectiveness of this intervention compared to other digital health modalities requires further investigation.

### 4.8. Future Directions

Given the exploratory nature of this pilot study, future research should focus on larger, multi-center randomized controlled trials to confirm the efficacy and cost-effectiveness of this metronome-cued walking mHealth system. Long-term follow-up studies are needed to assess the durability of the observed improvements and the optimal strategies for maintaining engagement. Additionally, comparative effectiveness studies evaluating this system against other digital health modalities, such as wearable-only interventions or synchronous tele-rehabilitation, would help position this approach within the broader landscape of pulmonary rehabilitation options. Exploring the potential mechanisms of metronome-cued walking—including whether benefits are mediated through improved gait efficiency, reduced dyspnea perception, or enhanced self-efficacy—would also provide valuable insights for intervention optimization.

## 5. Conclusions

In conclusion, this pilot study provides preliminary evidence that a personalized, home-based metronome-cued walking mHealth system may be associated with improvements in exercise capacity, dyspnea, health-related quality of life, and psychological well-being in patients with stable COPD. The program appeared safe in this small sample. The system addresses some barriers to center-based pulmonary rehabilitation and may offer a complementary strategy worth testing in future definitive trials.

## Figures and Tables

**Table 1:** Description of system applications.

System Component	Explanation
Assessment Report Module	At baseline, all participants undergo a comprehensive assessment in the hospital, including pulmonary function tests, CPET, 6MWT, and a series of questionnaires: the CAT, the mMRC, the HADS and the CCQ. Following the 12-week exercise intervention, participants are reassessed in the hospital using the 6MWT and the same set of questionnaires (CAT, mMRC, HADS and CCQ).
MCW Prescription Module	Participants in the intervention group (IG) are trained using a walking program based on the metronome-cued walking (MCW) system. This system is integrated into the mHealth application, guiding patients to walk at the speed set by the metronome. At the first visit, the optimal beat frequency for walking is determined based on the results of VO_2_ and 6MWD. The metronome rate provides rhythmic stimulation and is tailored for each patient using two calculations: (1) Walking speed on flat ground: Walking speed (m/min) = (VO_2_ − 3.5)/0.1 (2) Stride frequency per minute: Stride frequency = Walking speed/Step distance. The initial walking intensity is set at 70% of the maximum speed recorded during the 6MWT. Once the program starts, the metronome of the app guides the walking speed of the participants. Exercise intensity is then adjusted based on the modified Borg scale (0–10) to ensure appropriate progress and patient comfort.
Feedback Module	Pre-Walking Training: Before starting walking training, patients are instructed to record their heart rate, pulse oxygen saturation (SpO_2_), and any relevant symptoms, and upload these data daily to the mHealth system. Post-Walking Training: After completing each walking training session, patients are required to record their heart rate, pulse, oxygen saturation (SpO_2_), and symptoms, and upload the data daily to the mHealth system. Symptom Monitoring: Patients are encouraged to consistently report any symptoms experienced during or after training to facilitate continuous monitoring and evaluation.
Monitoring Module	Warning events are defined as any of the following situations: (1) SpO_2_ ≤ 88% while breathing room air. (2) Heart rate > 130 beats per minute. (3) Borg scale score ≥ 7. (4) Blood pressure exceeding 180/110 mmHg. (5) Symptoms such as angina, dizziness, or dyspnea after exercise training.

**Table 2:** Baseline demographic and clinical characteristics of the study participants.

Participant Demographics	CG (*n* = 12)	IG (*n* = 28)	t/Z	*p*
Age (years)	64.83 ± 8.43	66.46 ± 7.53	−0.61	0.548
BMI (kg/m^2^)	25.06 ± 2.37	24.99 ± 3.41	0.07	0.947
Pulmonary function				
FEV_1_ (L)	1.59 ± 0.49	1.47 ± 0.46	0.74	0.463
FEV_1_%pre (%)	54.33 ± 15.95	54.14 ± 14.37	0.04	0.971
FVC%pre (%)	85.75 ± 13.41	84.46 ± 16.28	0.24	0.811
Exercise tolerance				
6MWD (meters)	464.92 ± 56.22	460.57 ± 79.81	0.17	0.865
CPET characteristics				
Peak work rate (W)	98.07 ± 32.73	86.83 ± 26.50	−0.98	0.334
Peak VO_2_ (mL/kg/min)	14.43 ± 3.29	17.03 ± 4.49	−1.80	0.079
Breathing reserve	13.59 ± 7.66	14.25 ± 6.00	−0.30	0.769
Self-reported questionnaire				
CAT score	11.00 (8.25,13.75)	13.00 (12.00,17.00)	−1.62	0.106
CCQ score	0.95 (0.75,1.55)	1.15 (0.83,1.60)	−0.86	0.319
mMRC (grade)	2.00 (1.00,2.00)	1.00 (1.00,2.00)	−1.17	0.24
HAD-A score	1.00 (1.00,2.75)	2.00 (1.00,6.00)	−1.46	0.143
HAD-D score	1.00 (0.25,3.75)	4.00 (1.00,6.00)	−1.87	0.062
BODE Index	1.00 (1.00,2.00)	1.00 (1.00,1.75)	−0.41	0.679
Current medication, *n* (%)				
LAMA	5 (42%)	12 (43%)	−0.01	0.944
LAMA+LABA+ICS	7 (58%)	16 (57%)		
LTOT, n (%)			−0.01	0.927
Yes	2 (17%)	5 (18%)		
No	10 (83%)	23 (82%)		

Note: Data are presented as means ± standard deviation (SD) or median (Q1, Q3), and numbers or percentages are presented as *n* (%), unless otherwise specified. CG, control group; IG, intervention group; BMI, body mass index; FEV1, forced expiratory volume in 1 s; FVC, forced vital capacity; 6MWD, 6 min walking distance; CAT, COPD Assessment Test; CCQ, Clinical COPD Questionnaire; mMRC, modified British Medical Research Council; HAD, Hospital Anxiety and Depression Scale; BODE: Body mass index, Obstruction, Dyspnea, Exercise capacity; LAMA, long-acting muscarinic antagonist; LABA, long-acting β2-agonist; ICS, inhaled corticosteroid; LTOT, long-term oxygen therapy; PAH, pulmonary arterial hypertension; CPET, cardiopulmonary exercise testing.

**Table 3:** Comparison of 12-week 6MWD between the two groups before and after adjusting for confounders.

Outcome	IG (*n* = 28)	CG (*n* = 28)	Unadjusted Difference (*p*-Value)	Adjusted Mean Difference (95%CI) ^a^	Adjust *p*-Value	Partial Eta^2^
6MWD at 12 Weeks (m)	497.26 ± 3.50 ^b^	453.24 ± 5.36 ^b^	30.59 (*p* = 0.012)	+44.02 (30.99 to 57.05)	<0.001	0.573

Note: 6MWD, 6 min walking distance. ^a^ Adjusted for age, baseline FEV1% predicted, and baseline 6MWD using analysis of covariance (ANCOVA). ^b^ Values are presented as estimated marginal means ± standard error (SE) derived from the ANCOVA model. partial eta squared (where “2” is the superscript for squaring, indicating the statistical effect size).

**Table 4:** A 12-week personalized walking intervention yielded significant improvements across multiple outcomes.

Variables	Group	Baseline	12 Weeks	Change (MD, 95%CI)	Z	*p*
6MWD (meters)	IG (*n* = 28)	460.57 ± 79.81	495.89 ± 83.26	35.32 (27.84,42.80)	−7.07	<0.001
	CG (*n* = 12)	464.92 ± 56.22	456.42 ± 58.61	−8.5 (−17.52,0.52)		
CAT score	IG (*n* = 28)	13.00 (12.00,17.00)	10.50 (8.25,12.00)	−2.21 (−3.33,−1.10)	−2.92	0.004
	CG (*n* = 12)	11.00 (8.25,13.75)	12.00 (9.00,14.00)	0.33 (−0.86,1.52)		
CCQ score	IG (*n* = 28)	1.15 (0.83,1.60)	1.00 (0.53,1.78)	−0.13 (−0.31,0.05)	−2.69	0.007
	CG (*n* = 12)	0.95 (0.75,1.55)	1.10 (0.70,1.56)	0.10 (−0.01,0.21)		
mMRC (grade)	IG (*n* = 28)	1.00 (1.00,2.00)	0.00 (0.00,1.00)	−0.54 (−0.78,−0.29)	−2.45	0.014
	CG (*n* = 12)	2.00 (1.00,2.00)	2.00 (1.00,2.00)	0.00 (−0.38,0.38)		
HAD-A score	IG (*n* = 28)	2.00 (1.00,6.00)	1.50 (0.00,3.75)	−1.11 (−1.75,−0.46)	−3.34	0.001
	CG (*n* = 12)	1.00 (1.00,2.75)	1.50 (0.25,3.75)	0.92 (0.13,1.70)		
HAD-D score	IG (*n* = 28)	4.00 (1.00,6.00)	2.00 (0.00,4.75)	−1.18 (−1.85,−0.51)	−2.16	0.031
	CG (*n* = 12)	1.00 (0.25,3.75)	1.50 (0.00,3.00)	0.17 (−0.73,1.06)		
BODE Index	IG (*n* = 28)	1.00 (1.00,1.75)	1.00 (1.00,1.00)	−0.04 (−0.16,0.09)	−1.65	0.100
	CG (*n* = 12)	1.00 (1.00,2.00)	1.00 (1.00,2.00)	0.17 (−0.08,0.41)		

Note: Data are presented as means ± standard deviation (SD) or median (Q1, Q3) unless otherwise stated. The sample sizes were *n* = 28 in the IG and *n* = 12 in the CG. CG, control group; IG, intervention group; CI, confidence interval; 6MWD, 6 min walking distance; CAT, COPD Assessment Test; CCQ, Clinical COPD Questionnaire; mMRC, modified British Medical Research Council; HADS, Hospital Anxiety and Depression Scale; BODE, Body mass index, Obstruction, Dyspnea, Exercise capacity.

## Data Availability

The data presented in this study are available on request from the corresponding author; the data are not publicly available due to privacy and ethical restrictions.

## Abbreviations

The following abbreviations are used in this manuscript:

6MWD	6 min walk distance
COPD	chronic obstructive pulmonary disease
MCID	minimal clinically important difference
FEV1	forced expiratory volume in 1 s
FVC	forced vital capacity
CAT	COPD Assessment Test
mMRC	modified British Medical Research Council
HADS	Hospital Anxiety and Depression Scale
CCQ	Clinical COPD Questionnaire
HCPs	health care providers

## Appendix A

The mobile application consists of three main components: the Patient Application serves as the primary interface for patients, delivering the training program and collecting feedback data to ensure proper engagement and progress tracking; the Cloud Server acts as the central hub for data exchange and processing, facilitating seamless communication between the Patient Application and the Doctor Workstation; and the Doctor Workstation is mainly used for safety monitoring and adjusting prescriptions as needed to ensure that personalized and safe training is provided for each patient.

Screen 1 (Patient Application): 康复训练 (Rehabilitation Training); 今日任务(Today’s Tasks); 排行榜 (Leaderboard); 运动日志 (Exercise Log); 步行训练 (Walking Training).

Screen 2 (Doctor workstation): 通讯录 (Contacts); 医生 (Doctors); 患者 (Patients).

Screen 3 (Daily diary screen): 运动期监测 (Monitoring after exercise); 运动后心率血氧 (Post-exercise heart rate and oxygen saturation); 运动中不良反应 (Adverse reactions during exercise; 胸痛 (Chest pain); 呼吸困难 (Dyspnea); 大汗 (Profuse sweating); 面色苍白 (Pale complexion).

Screen 4 (Metronome-Cued walking screen): 速度70步/min (Speed: 70 steps/min); 继续/停止 (Continue/Stop); 暂停 (Pause).

Screen 5 (Borg Scale screen): 运动后Borg评分 (Post-exercise Borg Scale) rating from 完全没有(None) to 极度严重 (extremely severe).

Screen 6 (Assessment screen): 有氧能力 (Aerobic Capacity assessment form); 是否完成六分钟步行试验? (Is the 6-Minute Walk Test completed?); 六分钟步行距离 (6-Minute Walk Distance); 6分钟步行步长 (6-Minute Walk Step Length); 6MWT前后的心率 (Pre/Post-6MWT HR); 6MWT前后的血氧 (Pre/Post-6MWT SpO_2_); 6MWT前后的Borg评分 (Borg score before and after 6MWT).

Screen 7 (establishment prescription screen): 步行训练处方 (Walking Training prescription; 每日 (Daily: 1 time); 每周 (Weekly: 3 days); 配速 (Pace: 70 steps/min); 时长 (Duration: 30 min).

Screen 8 (warnin Monitory screen): 运动监督 (Exercise Supervision); 随访监督 (Follow-up Supervision); 预警 (Alert).
